# General practitioners’ adoption of generative artificial intelligence in clinical practice in the UK: An updated online survey

**DOI:** 10.1177/20552076251394287

**Published:** 2025-11-25

**Authors:** Charlotte Blease, Josefin Hagström, Carolina Garcia Sanchez, Anna Kharko, Brian McMillan, Jens Gaab, Emma Brulin, Cosima Locher, Maria Hägglund, Sara Riggare, Kenneth D Mandl

**Affiliations:** 1 Participatory eHealth and Health Data Research Group, Department of Women's and Children's Health, 8097 Uppsala University, Uppsala, Sweden; 2 Centre for Primary Care and Health Services Research, 5292 University of Manchester, Manchester, UK; 3 Clinical Psychology and Psychotherapy, Faculty of Psychology, 27209 University of Basel, Basel, Switzerland; 4 Unit of Occupational Medicine, 27106 Institute of Environmental Medicine, Karolinska Institutet, Stockholm, Sweden; 5 59561 Uppsala University Hospital, Uppsala, Sweden; 6 Boston Children's Hospital, 1811 Harvard Medical School, Boston, USA

**Keywords:** Generative AI, general practice, primary care, large language models, education, training, survey

## Abstract

**Background:**

Following the launch of ChatGPT in November 2022, interest in large language model-powered chatbots has soared with increasing focus on the clinical potential of these tools. Building on a previous survey conducted in 2024, we sought to gauge general practitioners’ (GPs) adoption of this new generation of chatbots to assist with any aspect of clinical practice in the UK.

**Methods:**

An online survey was disseminated in January 2025 to a stratified convenience sample of GPs registered with the clinician marketing platform Doctors.net.uk. The research was conducted as part of a scheduled monthly ‘omnibus survey,’ designed to achieve a fixed sample size of 1000 participants.

**Results:**

Of the 1005 respondents, 50% respondents were men, 54% were 46 years or older. 25% reported using generative artificial intelligence (GenAI) tools in clinical practice; of these, 35% reported using these tools to generate documentation after patient appointments, 27% to suggest a differential diagnosis, 24% for treatment options, and 24% for referrals. Of the 249 GPs who used generative AI for clinical tasks, 71% said that, in general, these tools reduced work burdens. In the last 12 months, 85% reported that their employer had not encouraged them to use GenAI tools, but only 3% said their employer had prohibited them from using GenAI tools in their work; 95% reported they had no professional training in using GenAI tools in their work.

**Conclusions:**

This survey suggests that doctors’ use of GenAI in clinical practice may be growing in the UK. Findings suggest that UK GPs may benefit from these tools, especially for administrative tasks and clinical reasoning support, and after adopting them, most users reported a decrease in work burdens. Continued absence of reported training remains a concern.

## Introduction

Since the release of ChatGPT in November 2022, there has been a surge in interest surrounding large language model (LLM)-powered chatbots, particularly in their potential applications within clinical settings. These chatbots are built on Generative Pre-trained Transformer (GPT) architectures, meaning they are pre-trained on vast datasets before being fine-tuned for specific applications. They generate responses based on probabilistic patterns learned during training, allowing them to produce coherent and contextually relevant text. Unlike traditional search engines, they not only summarize and generate text rapidly but also engage in conversational interactions and retain context from previous inputs.

A growing body of research highlights the potential of these models to support clinicians including (but not limited to) rapidly drafting medical documentation via “ambient” or so-called “listening” artificial intelligence (AI),^1,2^ assisting with writing accessible and empathetic clinical notes that patients might read,^3–5^ and assisting in formulating differential diagnoses.^6^ However, these tools are not without limitations.^7^ They are susceptible to generating inaccurate information (a phenomenon known as “hallucination”), and may reinforce existing biases related to race, gender, and disability in healthcare, contributing to “algorithmic discrimination”.^8^ Additionally, as consumer-facing technologies, they raise concerns about patient data privacy and security.^9,10^

However, most studies to date have focused on experimental settings rather than real-world use by key stakeholders. Scarce research has explored the extent to which doctors are adopting generative artificial intelligence tools (“GenAI”) into clinical workflows. Attempting to address this gap, in October 2023, a survey of 138 psychiatrists affiliated with the American Psychiatric Association found that 44% had used ChatGPT-3.5, and 33% had used GPT-4.0 to assist with clinical questions.^11^ While 70% agreed that GenAI could improve documentation efficiency, 75% believed most patients would consult AI before seeing a doctor, and 90% emphasized the need for better clinician training and support in understanding these tools.

In November 2023, another study, which gathered responses from 938 UK public sector professionals, including National Health Service (NHS) workers (24%), emergency service workers (15%), and others in education and social work, revealed similar findings.^12^ Among the respondents, 45% were aware of colleagues using GenAI, while 22% reported using it themselves. More recently, in February 2024, we surveyed 1006 general practitioners (GPs) in the UK about their use of generative AI in clinical practice.^13^ Among the respondents, 20% reported using GenAI tools, mainly for documentation (29%) and suggesting differential diagnoses (28%). Meanwhile, other studies, including findings emerging from the current study, have focused on GPs’ opinions about GenAI in healthcare.^11,14,15^

Despite the surging interest in GenAI in healthcare, research on clinicians’ adoption remains extremely limited. Furthermore, even while acknowledging that these tools are still relatively new, no studies have sought to track secular trends in adoption. Therefore, we investigated how UK General Practitioners (GPs) are integrating generative AI chatbots into daily practice. Building on our 2024 survey,^13^ we aimed to replicate the survey one year later, and to re-assess the extent to which GPs are using these tools for clinical tasks, to provide a clearer picture of their adoption trajectory.

The survey instrument used in this study was identical to that administered in 2024, enabling direct year-on-year comparisons. This approach was used to allow us to track changes in adoption rates and examine whether associations between GenAI use and key demographic and practice variables (e.g., gender, age, and practice size) have shifted over time. Contextual factors such as institutional support, training, and perceived usefulness—also included in the 2024 instrument—are presented here alongside 2025 data to provide a longitudinal perspective, while previously published analyses of these variables using 2024 data are referenced rather than repeated.

This repeated cross-sectional design represents, to our knowledge, the first national year-on-year survey of UK GPs’ use of GenAI tools. Such longitudinal surveillance is essential in the early adoption phase of new health technologies, offering timely insights for policymakers, educators, and regulators as they develop strategies to support safe, equitable, and effective integration into clinical workflows.

## Methods

### Study design and participant recruitment

Using a stratified convenience sample, we conducted an online survey, called the GPAI-UK-2025 Survey, with GPs registered with Doctors.net.uk, the largest professional network for UK doctors affiliated with the General Medical Council (GMC).^16^ Stratification was applied to achieve a sample broadly comparable by age and gender to the GMC Council Database. While this approach improved demographic representativeness, it remains a non-probability sample and may not fully reflect the characteristics or views of the wider UK GP population. Doctors.net.uk has 254,741 members, representing around 65% of the approximately 390,000 registered doctors in the UK.^17^ Among those registered with Doctors.net.uk, a variable percentage of GPs active within the community also consented to being sent survey invitations via email. The survey was part of a recurring monthly ‘omnibus survey’ with a predetermined sample size of 1000 participants, requiring respondents to answer all closed-ended questions. This sample reflects a fixed capacity offered by the omnibus survey design which is determined by the survey provider. Access to Doctors.net.uk is restricted to verified members, including GMC-registered doctors, UK medical students, and international medical graduates. Verification requires the provision of a valid GMC number or equivalent professional credentials, which are checked before account activation. For the present study, only verified GPs were invited to participate. While no additional fraud detection software was used beyond the platform's built-in authentication systems, these verification procedures ensured that all respondents were qualified medical professionals. Since only registered users had access to the survey, multiple responses by the same user were not possible.

Approximately 25,569 GPs were active online in the Doctors.net.uk community during the period when the survey was administered. A random sample of GPs was invited via email or through homepage advertisements on Doctors.net.uk, depending on their preferred method of survey notifications. The survey was open from 7 January 2025 to 26 January 2025 and closed after reaching 1005 responses.

Before launch, the survey underwent pretesting and a pilot phase with five UK GPs. The survey (see Appendix 1) was designed to take 3–5 min to complete. It adheres to the CROSS (Checklist for Reporting Of Survey Studies) (see Appendix 2).

This study used a repeated cross-sectional design, re-administering the same survey instrument to independent samples of UK GPs in 2024 and 2025, enabling year-on-year comparisons while acknowledging that individual respondents were not followed over time. To guide interpretation of adoption patterns, we drew on the Diffusion of Innovation (DoI) framework, a widely used model for understanding the spread of new technologies.^18^ DoI characterizes adopters as innovators, early adopters, early majority, late majority, or laggards, and examines how factors such as perceived advantage, compatibility with existing workflows, and organizational support influence uptake. This lens is particularly relevant for emerging health technologies such as generative AI, where adoption is still in the early stages and may be uneven across subgroups.

### Ethical considerations

The study received ethical approval from the Faculty of Psychology, University of Basel (# 030-24-1). All invited GPs were assured anonymity, and informed consent was obtained before participation. The survey was conducted via the secure platform Doctors.net.uk, which ensures that personal data remains encrypted and fully anonymized, with no linkage between responses and identifiable information. Email addresses and other personal identifiers were removed from respondent IDs before data was transferred to the research team. Doctors.net.uk complies with the European Union's General Data Protection Regulation (GDPR). To encourage participation, respondents received a £7.50 (US$8.80, €8.83) shopping voucher upon survey completion.

### Survey

The GPAI-UK-2025 Survey was divided into 18 items embedded in four sections (17 closed-ended questions and 1 open-ended question). In Section A, question 1, participants were asked whether they had ever purposely used GenAI to assist in any aspect of clinical practice. If participants answered negatively, they were requested to continue the survey at question 2; if participants answered affirmatively, they were requested to select which tools they had used from a list, to indicate what clinical tasks they had used these tools for, and to report whether, in general, they perceived these tools to reduce work burdens. In question 2, all participants were asked whether, in the last 12 months, their employer had encouraged them to use GenAI tools in their work, whether their employer had prohibited the use of these tools, and whether they had participated in any professional training provided by their employer on the use of these tools. All questions included mandatory closed-ended responses, including—where appropriate—yes/no/don’t know options. Therefore, no missing data were recorded in the dataset; consequently, no methods for handling missing data were necessary. Section B focused on GPs’ opinions about GenAI on various aspects of clinical practice; Section C included an optional open-comment question. Finally, Section D requested demographic information.

In this paper, we report exclusively on Section A and focus on GPs’ experiences with GenAI tools in clinical practice. As noted, findings on GPs’ opinions about GenAI in clinical practice (Section B) have already been published in this journal.^14^ In addition, a qualitative analysis of GPs’ free text responses (Section C) is published elsewhere.^19^

## Statistical analysis

Descriptive statistics were used to summarize GPs’ characteristics and their adoption and experiences of GenAI in clinical practice. Multivariable binary logistic regression (for dichotomous outcomes) and multivariable ordinal logistic regression (for ordinal outcomes) were conducted to assess associations between the outcome variables and gender, age, and practice size, adjusting for these factors as potential confounders. Chi-square tests were used to explore associations between use among groups based on prior encouragement from the employer, prohibition, or training. The response alternative “Prefer not to say” was excluded from the gender analysis due to a low number of responses (n = 13). A significance level of 0.05 (two-tailed) was applied to all analyses, which were conducted using SPSS version 28. Likewise, Python version 3.11.9 was used with the pandas, SciPy, and stats model packages for some analyses.

## Results

### Overview

In total, 1141 unique users accessed the opening page of the survey. This figure served as the denominator for calculating both viewing and participation metrics. Because all 1141 individuals had direct exposure via email invitations or website links, the view rate was 100%. Of these, 1067 agreed to take part by selecting “yes” to the consent question, corresponding to a participation rate of 94% (1067/1141 × 100). Of those who consented, 1005 completed every survey item, giving a completion rate of 94% (1005/1067 × 100). For reference, 1141 denotes the total number of unique visitors, 1067 the number who consented, and 1005 the number who completed the survey in full.

### Respondent characteristics

Of the total number of 1005 respondents, 506 (50%) were men. In terms of age distribution, 86 (9%) were 35 or younger, 373 (37%) were aged 36–45, 366 (36%) were aged 46–55, and 180 (18%) were 56 or older. Regarding professional roles, 439 (44%) reported being GP Partners/Principals, 380 (38%) were Salaried GPs, 150 (15%) were Locum GPs, and 36 (4%) were GP Registrars. 831 (81%) of GPs were from England (see Appendix 3) and 356 (35%) worked in a GP practice with 12,501 patients or more (see Table 1). Appendix 3 provides a comparison of respondents with the broader UK GP workforce.

**Table 1.** GP practice size.

Distribution of survey respondents by patient list size.	Total		
Patient list size	n	Percentage	95% CI
Up to 5000 patients	112	11%	9.2–13.1
5001–7500 patients	165	16%	14.2–18.9
7501–10,000 patients	202	20%	17.8–22.8
10,001–12,500 patients	170	17%	14.6–19.1
12,501 patients or more	356	35%	32.4–38.2
**Total**	1005	100%	

### GPs’ experiences with using generative AI

Of the 1005 respondents, 249 (25%) reported purposefully using GenAI tools in clinical practice. The overall uptake rate suggests that GenAI use among UK GPs is in the early adopter phase of the DoI curve, with the majority yet to integrate such tools into practice. Among those who confirmed usage and provided further details, Table 2 summarizes the adoption of specific GenAI models, while Table 3 outlines the clinical tasks for which these tools were used. Of the 25% (249) who reported using GenAI tools, 71% (176) reported that “in general,” these tools have helped reduce work burdens.

**Table 2.** Usage of different AI tools.

	Total among all GenAI users (n = 249)	
AI tool	t	Percentage %^a^(95%CI)
ChatGPT	176	71% (65.1–76.3)
Microsoft Copilot	41	16% (12.0–20.9)
Bing AI	14	6% (2.8–8.4)
Bard	11	4% (2.0–6.8)
Claude	6	2% (0.8–4.4)
Med-PaLM	2	1% (0–2.0)
Other (please specify) *Included (among others)*	82	33% (27.3–39.0)
* *· *Heidi Health*	44	18%
* *· *Gemini*	8	3%
· *Ankit Kant*	4	2%

a Since survey items requested participants to select all options that applied, % does not total 100.

**Table 3.** Purposes for using generative AI tools.

	Total among all GenAI users (n = 249)	
Purpose	N	Percentage %^a^
Documentation after patient appointments	87	35%
Differential diagnoses	67	27%
Treatment options	61	24%
Referrals	59	24%
Patient summarization/timelines from prior documentation	37	15%
Medical certification e.g., for employment	18	7%
Other (please specify)	66	27%
*Included*		
· *Drafting letters*	11	7%
· *Responding to complaints*	6	4%
· *Creating presentations or teaching material*	6	4%
*· Triage*	3	2%

a Since survey items requested participants to select all options that applied, % does not total 100.

### GPs’ employment experiences with generative AI

Participants were asked about their employer's stance on GenAI over the past 12 months. The majority, 85% (850), reported that their employer had not encouraged the use of GenAI tools, while 11% (107) said they had, and 5% (48) were uncertain. Similarly, 91% (913) stated that their employer had not prohibited the use of GenAI, whereas 3% (35) reported restrictions, and 6% (57) were unsure. When asked about participation in employer-provided training on GenAI, 95% (953) indicated they had not received any, while only 5% (52) had.

### Factors associated with GPs’ experiences with generative AI

Multivariable logistic regression indicated that while younger age and larger size of practice were significantly associated with the use of GenAI tools, gender was not. Of the variables explored, only age was related to views on GenAI tools reducing work burdens, with younger GPs having higher odds of reporting that these tools reduced their work burdens. A comparison of the distribution of age, gender, location, and practice size is further presented in Appendix 4.

Among the participants reporting having used GenAI tools, gender was the variable most commonly associated with the purpose of using these tools: male GPs were more likely to have used them for documentation after patient appointments, differential diagnosis, and referrals compared to their female counterparts. A significant association was also found between larger practice size and using GenAI tools for differential diagnosis.

While perceived improvements were not related to practice size, ordinal regression showed that male and older GPs were more likely to believe that GenAI tools would improve their work related to diagnostic accuracy. Furthermore, male GPs perceived improvement of GenAI on several additional aspects of their work: creation of personalized treatment plans, conveying empathy, and patient communication. While older GPs perceived improvement in prognostic accuracy, younger GPs were more likely to believe that GenAI tools would improve documentation.

All variables were significantly related to the potential effects on healthcare of GenAI tools. Compared to others, GPs who were female, older, or working at a larger practice were more likely to believe that GPs need more support/training in understanding GenAI tools. Male GPs were more likely to believe that GenAI tools would increase patient privacy and decrease patient harm, where the latter was also perceived by larger practice GPs. Plus, while GPs in larger practices believed that GPs could increase efficiencies, those in smaller practices anticipated an increase in errors. Lastly, higher age was related to believing GenAI tools would increase inequities in care delivery.

Chi-square tests were conducted on the sample who reported their gender (N = 992) to assess whether GenAI use was based on having been encouraged, prohibited, or received training. GPs demonstrated higher use of GenAI tools among those who had been: encouraged to use (χ²(1, n = 946) = 60.193, *p* < 0.001), prohibited from using (χ²(1, n = 938) = 5.869, *p* = 0.015), or received training (N = 992, *p* < 0.001) in using AI tools in their work.

## Discussion

### Principal findings

This is one of the few surveys to date on the adoption of GenAI tools in healthcare. Among the 1005 participating GPs, 25% (249) reported using GenAI in clinical practice, with key applications including the creation of documentation after patient appointments (35%), differential diagnosis (27%), treatment suggestions (24%), and referrals (24%). A substantial 71% (176) of users reported that GenAI helped reduce their workload, highlighting its potential to mitigate burnout and improve efficiency in clinical settings. A number of associations were identified, including that male GPs were more likely to have used GenAI tools for the purpose of differential diagnosis and referrals. Furthermore, younger GPs were more likely to report that these tools reduced their work burdens.

Reported institutional support for GenAI adoption remained low among our participants. The majority—85% (850)—of respondents stated that their employer had neither encouraged nor discouraged GenAI use, while only 3% (35) reported an outright prohibition. Notably, 95% (953) of GPs indicated they had received no professional training on the use of these tools, despite their increasing adoption. Relatedly, and worryingly, those who reported that they had been prohibited from using or received training reported higher use. Notably, adoption of the commercially available tool ChatGPT was the highest, with 71% (176) of GenAI users adopting it for clinical tasks. These findings underscore both the potential benefits of GenAI in streamlining administrative tasks and the urgent need for structured guidance and training to ensure safe, ethical, and effective implementation.

Framing these results within the DoI model suggests that GenAI adoption in UK primary care remains in its early stages, led by a subset of innovators and early adopters.^18^ Understanding the characteristics of these groups—such as younger age or working in larger practices—may help target interventions that encourage uptake among the early majority, thereby accelerating safe and effective integration.

### Comparison with previous literature

Our findings align with and build upon prior research examining GenAI adoption in healthcare. Compared with our 2024 survey,^13^ which found a 20% adoption rate, our results suggest a modest increase over one year. While the magnitude of this change is limited, tracking such trends in the early diffusion period of new technologies is critical for anticipating adoption plateaus, identifying lagging subgroups, and informing targeted interventions. As noted earlier, in the 2024 survey found that GenAI was primarily used for documentation (29%) and differential diagnosis (28%).^13^ Comparison with our 2024 survey^14^ indicates modest changes in who uses GenAI too. While younger GPs continue to report the highest uptake, adoption has risen slightly among older age groups, suggesting early diffusion beyond the youngest cohort. The gender gap observed in 2024 has narrowed, with a proportionally larger increase in reported use among female GPs. The association between practice size and adoption remains, with higher uptake in larger practices, but appears more pronounced than in 2024, potentially reflecting the role of organizational resources and peer influence in supporting adoption. These patterns are consistent with the early adopter phase described in the DoI model and underscore the importance of targeting interventions to facilitate uptake among groups that remain underrepresented in current usage patterns.

As noted, in our present study, male and female GPs used GenAI at similar rates, diverging from broader trends that indicate a persistent gender gap in AI adoption across most regions, sectors, and occupations, with men generally using these tools more than women.^20^ Notably, we observed a higher use of GenAI for differential diagnosis and referrals, though the underlying reasons remain unclear. Further investigation is needed to explore whether this reflects differences in clinical workflow preferences, confidence in AI-assisted decision-making, or other contextual factors influencing AI adoption in medical practice.

### Regulation and training

GenAI tools have a unique capacity to elicit anthropomorphism—the tendency to attribute human-like qualities to machines—due to their dialogic nature and compelling, context-aware responses. This makes them particularly effective at extracting information, as users may unknowingly over-disclose personal details, perceiving the AI as an intentional or trustworthy interlocutor. Acknowledging the novel challenges with these tools, the European Union's AI Act—globally, the most robust legislation on these technologies—classifies consumer GenAI tools as “high risk”, citing concerns related to cybersecurity, accuracy, reliability (robustness), and transparency.^21^ In short, using these tools for clinical tasks raises grave concerns about patient safety, and privacy in relation to sensitive health information.

In the UK, the Medicines and Healthcare products Regulatory Agency (MHRA) notes that if a developer claims an AI product can be used in a medical setting, this would most likely require the product to be registered as a medical device^22^ with the stringent quality assurance criteria this entails. Most LLMs, however, do not claim to be suitable for clinical use, and the GMC recommends that clinicians “use their professional judgement to apply the principles in our guidance to the use of innovative technologies or AI tools”,^19^ in line with regulators from comparable jurisdictions.^23^ Both the British Medical Association (BMA) and the Royal College of General Practitioners (RCGP) have highlighted the need for stringent regulation to ensure the safety and security of AI tools for clinical use^.^^24,25^

In recognition that AI is increasingly being used by UK clinicians, online training has been made available via the NHS Learning Hub,^26^ and clinicians can now become certified as an ‘Artificial Intelligence Practitioner.’ In response to the increasing use of AI-enabled ‘digital scribes’ in GP settings and beyond, NHS England has also recently provided detailed guidance on the use of such tools in healthcare settings.^27^ There are now also postgraduate awards aimed specifically at healthcare professionals providing training on the use of AI in clinical contexts.^28,29^ A recent survey of 175 students from 19 UK medical schools found that the majority of undergraduate medical students were not aware of any dedicated teaching on the topic of data science and AI in their curriculum,^30^ and another UK study of 210 trainee doctors found that 92% felt the AI training they received was insufficient.^31^ In light of these findings, the Medical Schools Council has stressed that it is essential for medical students to be taught how to use artificial intelligence (AI) as part of their training and has developed a set of proposed learning competencies for this purpose.^23^

Despite growing use of GenAI, most GPs reported no employer encouragement or training, likely reflecting institutional caution or uncertainty amid regulatory ambiguities, and unknown clinical risk. Yet, our data show that both encouragement and training are significantly associated with higher adoption. Structured, evidence-based training, such as NHS-certified modules or CME-integrated content, may be key to bridging the gap between interest and safe clinical use.

### Survey strengths and limitations

This is one of the few surveys illuminating the use of GenAI among GPs in the UK, which is its main strength. However, this study also has several limitations that must be acknowledged. Firstly, the non-probability sampling method limits the generalizability of the findings. Doctors.net.uk is a widely used professional platform that reaches a significant number of UK GPs. Our sample was representative of the UK GP population when considering age and region of practice based on the GMC Registry (see Appendix 3). However, our sample had an equal gender split among respondents, while nationally, there are more women than men (58% vs 42%, respectively). This may have influenced some findings, for example, the effect of gender on the use of GenAI, including for specific tasks.

Meanwhile, it is conceivable that those who do not engage with online medical communities, are not on Doctors.net.uk, or are less active online in their daily work were not represented by our survey. Although stratified sampling was used to approximate the national GP age and gender distribution, the non-probability nature of the sample means self-selection bias remains possible. Respondents may be more digitally engaged, or more interested in GenAI than the wider GP population. These factors could limit the generalizability of our findings to GPs who are less familiar with, or less inclined to use, digital tools in their clinical practice.

The survey results may have been influenced by responder bias, particularly if participants who were already inclined to use GenAI were more likely to respond. This may lead to an overrepresentation of AI users in the study sample, and the true number may be lower. This study relied entirely on self-reported data, which cannot be independently verified and may be subject to recall or social desirability bias; future research should triangulate self-report with objective measures such as system usage logs, electronic health record integration data, or qualitative interviews to validate and contextualize reported usage patterns.

During the statistical analysis, several chi-square tests were conducted: as a result, there is an increased risk of Type I error, and caution should be exercised when interpreting the results. Finally, while our central concern was with GPs’ GenAI adoption and usage patterns, we emphasize that we did not evaluate whether these tools improved diagnostic accuracy, treatment outcomes, or patient satisfaction.

### Future research directions

Future studies could build on this longitudinal foundation by incorporating predictive modelling to forecast adoption trajectories, qualitative research to capture in-depth perspectives from both early and late adopters, and comparative international analyses to contextualize UK trends. Given the increasing adoption of GenAI in primary care, future research should focus on several key areas. First, studies should assess how AI influences clinical decision-making and patient outcomes, moving beyond self-reported efficiency improvements to measurable clinical benefits. Controlled trials could compare AI-assisted and non-AI-assisted diagnostic accuracy, treatment planning, and patient engagement.

Second, research should explore the ethical and regulatory challenges associated with AI integration in clinical practice. Issues such as data privacy, bias in AI-generated recommendations, and medico-legal responsibility require urgent attention. Investigating how these concerns affect GP adoption rates and trust in AI will be essential for developing appropriate guidelines.

Third, training interventions should be developed and evaluated. With 95% of surveyed GPs reporting no formal training, structured educational programs tailored to different levels of AI proficiency should be implemented and assessed.^26^ Future studies could examine the effectiveness of AI training programs in improving clinical safety, reducing errors, and enhancing the overall integration of GenAI into practice.

Finally, we recommend that further research should focus on doctors’ ongoing use of these tools, employing qualitative methodologies to capture a more nuanced understanding of their experiences and perspectives. Additionally, exploring patients’ experiences with GenAI in their care—including their perceptions of and trust in doctors who use these tools—is essential.

## Conclusion

This study provides crucial insights into the growing adoption of GenAI among UK GPs. While 25% reported using GenAI tools, primarily for documentation and administrative support, the lack of formal training and institutional guidance remains a significant barrier. Despite potential benefits in reducing workload and streamlining tasks, AI adoption in clinical settings continues to occur in an unsupervised and fragmented manner.

Building on previous research, our findings suggest that AI use among GPs is increasing, yet without structured policies, training, and oversight, its full potential may not be realized. To ensure safe, ethical, and effective AI integration, medical organizations and policymakers must develop guidelines, provide training, and establish clear regulatory frameworks. As GenAI evolves, its role in healthcare will depend not only on technological advancements but also on how well the medical community adapts to and governs its use.

## Supplemental Material

sj-docx-1-dhj-10.1177_20552076251394287 - Supplemental material for General practitioners’ adoption of generative artificial intelligence in clinical practice in the UK: An updated online survey Supplemental material, sj-docx-1-dhj-10.1177_20552076251394287 for General practitioners’ adoption of generative artificial intelligence in clinical practice in the UK: An updated online survey by Charlotte Blease, Josefin Hagström, Carolina Garcia Sanchez, Anna Kharko, Brian McMillan, Jens Gaab, Emma Brulin, Cosima Locher, Maria Hägglund, Sara Riggare and Kenneth D Mandl in DIGITAL HEALTH

sj-docx-2-dhj-10.1177_20552076251394287 - Supplemental material for General practitioners’ adoption of generative artificial intelligence in clinical practice in the UK: An updated online survey Supplemental material, sj-docx-2-dhj-10.1177_20552076251394287 for General practitioners’ adoption of generative artificial intelligence in clinical practice in the UK: An updated online survey by Charlotte Blease, Josefin Hagström, Carolina Garcia Sanchez, Anna Kharko, Brian McMillan, Jens Gaab, Emma Brulin, Cosima Locher, Maria Hägglund, Sara Riggare and Kenneth D Mandl in DIGITAL HEALTH

sj-docx-3-dhj-10.1177_20552076251394287 - Supplemental material for General practitioners’ adoption of generative artificial intelligence in clinical practice in the UK: An updated online survey Supplemental material, sj-docx-3-dhj-10.1177_20552076251394287 for General practitioners’ adoption of generative artificial intelligence in clinical practice in the UK: An updated online survey by Charlotte Blease, Josefin Hagström, Carolina Garcia Sanchez, Anna Kharko, Brian McMillan, Jens Gaab, Emma Brulin, Cosima Locher, Maria Hägglund, Sara Riggare and Kenneth D Mandl in DIGITAL HEALTH

sj-docx-4-dhj-10.1177_20552076251394287 - Supplemental material for General practitioners’ adoption of generative artificial intelligence in clinical practice in the UK: An updated online survey Supplemental material, sj-docx-4-dhj-10.1177_20552076251394287 for General practitioners’ adoption of generative artificial intelligence in clinical practice in the UK: An updated online survey by Charlotte Blease, Josefin Hagström, Carolina Garcia Sanchez, Anna Kharko, Brian McMillan, Jens Gaab, Emma Brulin, Cosima Locher, Maria Hägglund, Sara Riggare and Kenneth D Mandl in DIGITAL HEALTH
