# Supplementary material for: General practitioners’ adoption of generative artificial intelligence in clinical practice in the UK: An updated online survey
Source: Digit Health. 2025 Nov 25;11:20552076251394287. doi: 10.1177/20552076251394287 (PMC12647557; doi:10.1177/20552076251394287)
Supplement: sj-docx-3-dhj-10.1177_20552076251394287 - Supplemental material for General practitioners’ adoption of generative artificial intelligence in clinical practice in the UK: An updated online survey [file sj-docx-3-dhj-10.1177_20552076251394287.docx]

**Appendix 3**. Comparison of Study Sample with the General Medical Council (GMC) Registry

**Table 1.** Gender.

|  | **Sample**  (n = 992) | **GMC Registry**  (n = 70,403) | Chi-square Analysis |
| --- | --- | --- | --- |
|  |  |  | χ² (1) = 31.06, *p* < .001, Cramer’s V = .021 |
| **Woman** | 486 (48%) | 40,725 (58%) |  |
| **Man** | 506 (50%) | 29,678 (42%) |  |

*Note*: 13 (1%) participants responded *‘Prefer not to say’*, and their responses were excluded from the analysis.

**Table 2.** Age range.

|  | **Sample**  (n = 1,005) | **GMC Registry**  (n = 70,403) |
| --- | --- | --- |
| **35 years or younger** | 86 (9%) | 7,638 (11%) |
| **36 – 45 years** | 373 (37%) | 26,275 (37%) |
| **46 – 55 years** | 366 (36%) | 20,804 (30%) |
| **56 years or older** | 180 (18%) | 15,686 (22%) |

*Note*: Age ranges in the GMC Registry do not match the ones in the survey, so a statistical comparison was not carried out.

**Table 3.** Location.

|  | **Sample**  (n = 1,005) | **GMC Registry**  (n = 70,403) | Chi-square Analysis |
| --- | --- | --- | --- |
|  |  |  | χ² (9) = 13.54, *p* = .14, Cramer’s V = .014 |
| **England** |  |  |  |
| North West | 123 (12%) | 7,618 (11%) |  |
| North East and Yorkshire | 135 (13%) | 8,460 (12%) |  |
| Midlands | 154 (15%) | 10,386 (15%) |  |
| East of England | 84 (8%) | 5,982 (8%) |  |
| London | 115 (11%) | 10,351 (15%) |  |
| South East | 131 (13%) | 9,319 (13%) |  |
| South West | 89 (9%) | 6,685 (10%) |  |
| **Northern Ireland** | 34 (3%) | 2,025 (3%) |  |
| **Scotland** | 91 (9%) | 6,633 (9%) |  |
| **Wales** | 49 (5%) | 2,944 (4%) |  |

**Table 4.** Comparison of sociodemographic characteristics of GenAI users and non-users GPs.

|  | **Users**  (n = 249) | **Non-users**  (n =756 ) | Chi-square Analysis | FDR p-value |
| --- | --- | --- | --- | --- |
| **Gender*** |  |  | χ² (1) = 0.34, *p* < .557, Cramer’s V = .018 | *p* < .55 |
| Woman | 117 (24%) | 369 (76%) |  |  |
| Man | 131 (26%) | 375 (74%) |  |  |
| **Age** |  |  | χ² (3) = 6.91, *p* < .074, Cramer’s V = .082 | *p* < .099 |
| 35 years or under | 25 (29%) | 61 (71%) |  |  |
| 36 – 45 years | 102 (27%) | 271 (73%) |  |  |
| 46 – 55 years | 90 (25%) | 276 (75%) |  |  |
| 56 years or over | 32 (18%) | 148 (82%) |  |  |
| **Location** |  |  | χ² (3) = 12.07, *p* < .007, Cramer’s V = .109 | *p* < .028 |
| England | 222 (27%) | 609 (73%) |  |  |
| Northern Ireland | 8 (24%) | 26 (76%) |  |  |
| Scotland | 10 (11%) | 81 (89%) |  |  |
| Wales | 9 (18%) | 40 (82%) |  |  |
| **Practice Size** |  |  | χ² (4) = 11.17, *p* < .024, Cramer’s V = .105 | *p* < .049 |
| Up to 5,000 patients | 16 (14%) | 96 (86%) |  |  |
| 5,001-7,500 patients | 35 (21%) | 130 (79%) |  |  |
| 7,501-10,000 patients | 51 (25%) | 151 (75%) |  |  |
| 10,001-12,500 patients | 51 (30%) | 119 (70%) |  |  |
| 12,501 patients or more | 96 (14%) | 260 (86%) |  |  |

*Note*: All analyses are based on the whole sample (*n* = 1,005), except for *Gender* (*n* = 993), where responses *‘Prefer not to say’* (*n* = 13) were omitted.
