# Supplementary material for: General practitioners’ adoption of generative artificial intelligence in clinical practice in the UK: An updated online survey
Source: Digit Health. 2025 Nov 25;11:20552076251394287. doi: 10.1177/20552076251394287 (PMC12647557; doi:10.1177/20552076251394287)
Supplement: sj-docx-4-dhj-10.1177_20552076251394287 - Supplemental material for General practitioners’ adoption of generative artificial intelligence in clinical practice in the UK: An updated online survey [file sj-docx-4-dhj-10.1177_20552076251394287.docx]

**Appendix 4.** Regression analyses of factors associated with GPs’ Experiences with Generative AI

**Table 1**. Binary logistic regression analysis of factors associated with GenAI use in clinical practice, use purpose, and perceived reduced work burden.

| **Item** | | **Variable (OR [95% CI], *p*)** | | |
| --- | --- | --- | --- | --- |
|  | | **Gender** | **Age** | **Practice size** |
| *Used GenAI in clinical practice* | | 0.85, [0.63,1.14], .272 | **0.81, [0.69,0.96], .015** | **1.15, [1.03,1.28], .010** |
| *For what purpose(s) have you used GenAI tools?* | | | | |
|  | Documentation after patient appointments | **0.54 [0.30,0.98], .042** | 0.84 [0.60,1.18], .323 | 0.96 [0.77,1.20], .738 |
|  | Differential diagnosis | **0.39 [0.21,0.74], .004** | 1.04 [0.73,1.48], .837 | **1.28 [1.00,1.63], .050** |
|  | Treatment options | 0.82 [0.48,1.40], .462 | 0.74 [0.54,1.02], .068 | 1.11 [0.90,1.37], .316 |
|  | Referrals | **0.41 [0.19,0.88], .023** | 0.84 [0.55,1.28], .426 | 1.11 [0.84,1.48], .464 |
|  | Patient summarization/timelines from prior documentation | 0.68 [0.25,1.85], .452 | 1.01 [0.57,1.78], .980 | 0.90 [0.63,1.30], .579 |
|  | Medical certification e.g., for employment | 0.66 [0.36,1.21], .177 | 0.76 [0.53,1.08], .122 | 1.01 [0.80,1.28], .904 |
| *Reduced work burden* | | 0.84 [0.47,1.47], .534 | **0.69 [0.49,0.97], .035** | 1.05 [0.85,1.30], .658 |

*Note:* Gender was coded as 1 = Male, 2 = Female (reference). Age and practice size were treated as ordinal variables with higher categories indicating older age and larger practice size, respectively.

**Table 2**. Ordinal regression analysis of factors associated with the belief that GenAI tools will improve or change clinical practice.

| **Item** | | **Variable (B [95% CI], *p*)** | | |
| --- | --- | --- | --- | --- |
|  | | **Gender** | **Age** | **Practice size** |
| *I believe that GenAI tools will improve…* | | | | |
|  | Patient information gathering | 0.18 [-0.08,0.44], .178 | 0.06 [-0.09,0.21], .412 | 0.08 [-0.15,0.17], .101 |
|  | Diagnostic accuracy | **0.39 [0.12,0.66], .004** | **0.17 [0.02,0.32], .028** | 0.07 [-0.02,0.17], .141 |
|  | The creation of personalized treatment plans | **0.30 [0.04,0.57], .024** | 0.12 [-0.03,0.27], .106 | 0.03 [-0.06,0.13], .523 |
|  | Prognostic accuracy | 0.25 [-0.02,0.52], .068 | **0.15 [0.00,0.31], .047** | 0.00 [-0.09,0.10], .930 |
|  | Conveying empathy | **0.40 [0.15,0.66], .002** | -0.04 [-0.18,0.11], .637 | -0.05 [-0.14,0.04], .238 |
|  | Documentation | 0.05 [-0.20,0.30], .700 | **-0.16 [-0.30,-0.01], .036** | 0.09 [-0.01,0.18], .066 |
|  | Patient communication | **0.51 [0.26,0.77], <.001** | -0.10 [-0.24, 0.05], .178 | 0.04 [-0.05,0.13], .355 |
|  | Communication with other healthcare providers | 0.09 [-0.18,0.35], .512 | **-**0.15 [-0.30,0.00], .054 | 0.05 [-0.05,0.14], .348 |
| *I believe that GenAI tools will…* | | | | |
|  | Increase errors | -0.05 [-0.32,0.23], .750 | -0.04 [-0.20,0.16], .610 | **-0.10 [-0.20,0.00], .047** |
|  | Decrease patient harm | **0.49 [0.21,0.77], <.001** | 0.04 [-0.12,0.20], .633 | **0.11 [0.01,0.22], .027** |
|  | Increase patient privacy | **0.40 [0.13,0.68], .004** | -0.12 [-0.28,0.04], .138 | 0.04 [-0.06,0.13], .471 |
|  | Increase inequities in care delivery | -0.16 [-0.44,0.11], .246 | **0.19 [0.04,0.35], .016** | 0.03 [-0.07,0.13], .510 |
|  | Mean more patients will rely on AI tools instead of seeking medical attention | 0.02 [-0.23,0.28], .854 | 0.06 [-0.09, 0.20], .461 | 0.02 [-0.08,0.11], .746 |
|  | Mean GPs need more support/training in understanding them | **-0.50 [-0.75,-0.25], <.001** | **0.19 [0.05,0.33], .009** | **0.11 [0.02,0.20], .015** |
|  | Increase efficiencies in healthcare | 0.14 [-0.13,0.40], .311 | -0.13 [-0.28,0.02], .094 | **0.11 [0,02,0.20], .020** |

*Note:* Gender was coded as 1 = Male, 2 = Female (reference). Age and practice size were treated as ordinal variables with higher categories indicating older age and larger practice size, respectively.
